# Tietze’s Syndrome Post-COVID-19 Infection in an Adult Patient

**DOI:** 10.7759/cureus.27499

**Published:** 2022-07-31

**Authors:** Charlene Tan, Rachel Lim, Marcus Yeow, Jeffrey Fong, Tharmmambal Balakrishnan

**Affiliations:** 1 Internal Medicine, Singapore General Hospital, Singapore, SGP; 2 Diagnostic Radiology, Singapore General Hospital, Singapore, SGP

**Keywords:** tietze syndrome, post-covid-19, tietze’s syndrome, costochondritis, chest pain, covid-19

## Abstract

Tietze’s syndrome is a rare inflammatory disorder characterized by chest well swelling and inflammation of the costal cartilages. We describe a gentleman with repeated presentations to the emergency department (ED) with left-sided chest and sternoclavicular pain on a background of recent asymptomatic coronavirus disease 2019 (COVID-19) infection. He had elevated inflammatory markers and MRI subsequently confirmed the diagnosis of Tietze’s syndrome. Anti-inflammatory medications and colchicine eventually led to a complete resolution of symptoms. This case highlights how Tietze’s syndrome -- a disorder that is potentially self-limiting, can cause great distress and should be a differential diagnosis of chest pain after excluding life-threatening etiologies related to COVID-19.

## Introduction

Costochondritis occurs due to inflammation of the costal cartilages and presents with chest wall pain. It is often incorrectly interchangeably used with Tietze’s syndrome; a rare inflammatory disorder distinguished by additional chest wall swelling. These benign conditions are often reproducible on palpation [1]. The exact cause is often unknown, although it has been associated with chronic excessive coughing, vomiting, trauma to the chest as well as respiratory infections particularly viral. Coronavirus disease 2019 (COVID-19)-related costochondritis has been reported among children with complete resolution using nonsteroidal anti-inflammatory drugs (NSAIDs) and colchicine [2]. Early recognition and awareness are important following exclusion of other cardiac and pulmonary complications especially in COVID-19 patients, as symptoms can be distressing, resulting in repeated admissions. We report a case of a man who presented with left-sided chest and sternoclavicular pain and had confirmed radiologic features of Tietze’s syndrome. This is on the background of full COVID-19 vaccination including booster shot and recent asymptomatic infection.

## Case presentation

A 44-year-old male lorry driver first presented to the emergency department (ED) with symptoms of sudden onset of the left-sided chest and sternoclavicular pain radiating to his left neck while driving. The pain was sharp with a pain score 7/10 and without associated cardiopulmonary or neurological symptoms or precipitant.

He had a past medical history of gout and hyperlipidemia which were managed with lifestyle modifications. He was not on any long-term medications, had no history of any other cardiovascular disease or any significant family history, and had a negative treadmill exercise stress test two years ago. He had infrequent gout attacks affecting his knees.

Initial investigations of troponin-T, renal panel, electrocardiogram, and chest radiograph were unremarkable. However, a routine COVID-19 polymerase chain reaction (PCR) test returned positive with a high cycle threshold (CT) value of 43.0 for N2 protein indicating recovery. He was discharged with analgesia (paracetamol/orphenadrine and tramadol) with a diagnosis of musculoskeletal pain. As the symptoms persisted, he re-presented to ED with the same severity and again was discharged with additional analgesia etoricoxib after a few tests. The initial impression of musculoskeletal pain remained unchanged after the second ED presentation. The pain increased in severity to a pain score of 10/10 over the next two days and required admission.

On clinical examination, his vitals were normal. The lateral range of movement of his left shoulder was, however, limited to 90 degrees due to the pain. Point tenderness was maximal over the left sternoclavicular joint with soft tissue swelling noted. The neurovascular examination of his upper limbs was normal. The cardiac and respiratory examination was otherwise unremarkable.

Investigations revealed normal hemoglobin, platelet, kidney, electrolyte, liver panel, serum uric acid, and creatine kinase including repeated troponin-T levels and d-dimer. Inflammatory markers were elevated with C-reactive protein 105 mg/L (range 0.2-9.1 mg/L), leucocyte count 11.0 (range 4.0-10 x 109/L), and erythrocyte sedimentation rate 61 mm/h (range 1-10 mm/h). Laboratory findings are shown in Table 1.

**Table 1:** Laboratory values of investigations performed. WBC, white blood cell count; MCV, mean corpuscular volume; ESR, erythrocyte sedimentation rate; CRP, C-reactive protein; FEU, fibrinogen equivalent units

Test	Result	Normal range
WBC (x10^9^/L)	11.09	4-10
Neutrophils (%)	85.9	40-75
Lymphocytes (%)	7.8	15-41
Hemoglobin (g/dL)	15.3	14-18
MCV (fL)	88.4	78-98
Platelet count (x10^9^/L)	384	140-440
ESR (mm/h)	61	1-10
Albumin (g/L)	38	40-51
Urea (mmol/L)	3.2	2.7-6.9
Creatinine (umol/L)	63	54-101
Sodium (mmol/L)	138	136-146
Potassium (mmol/L)	4.8	3.6-5.0
Chloride (mmol/L)	102	100-107
Bicarbonate (mmol/L)	24.8	19-29
Total calcium (mmol/L)	2.26	2.09-2.46
Phosphate (mmol/L)	1.50	0.94-1.5
CRP (mg/L)	105	0.2-9.1
D-dimer (mg/mL FEU)	Below limit of detection	0.19-0.55
Troponin (ng/L)	<13	<30
Uric acid (umol/L)	459	218-578
Creatine kinase (U/L)	69	56-336
Bilirubin (umol/L)	15	7-32
Alkaline phosphatase (U/L)	87	39-99
Alkaline transaminase (U/L)	19	6-66
Aspartate transaminase (U/L)	16	12-42
Gamma-glutamyl transferase (U/L)	68	14-94

Radiographs of his chest, left sternoclavicular joint, and scapula ruled out fractures, dislocations, and destructive lesions. Electrocardiogram was unremarkable.

**Figure 1:**
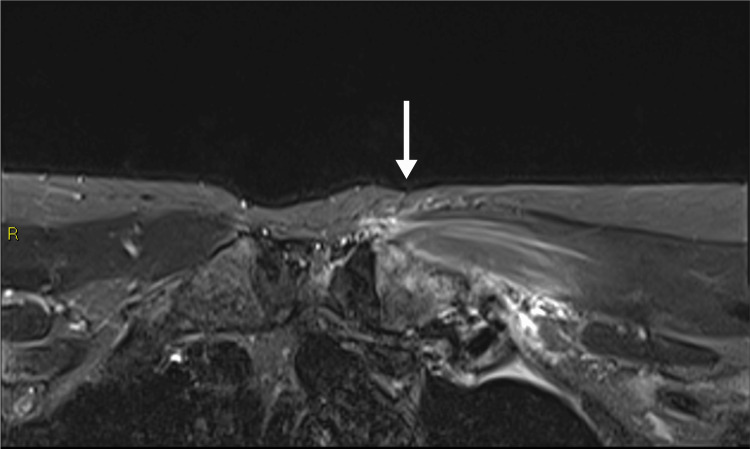
Axial fat suppressed fluid-sensitive MRI shows edema in the left distal first rib and surrounding soft tissue, in keeping with costochondritis. There is also mild signal change in the adjacent medical clavicle head. There is neither significant effusion of the left sternoclavicular joint, nor is there marrow edema in the sternum. The overlying pectoralis major muscle is edematous, likely secondary to reactive change.

**Figure 2:**
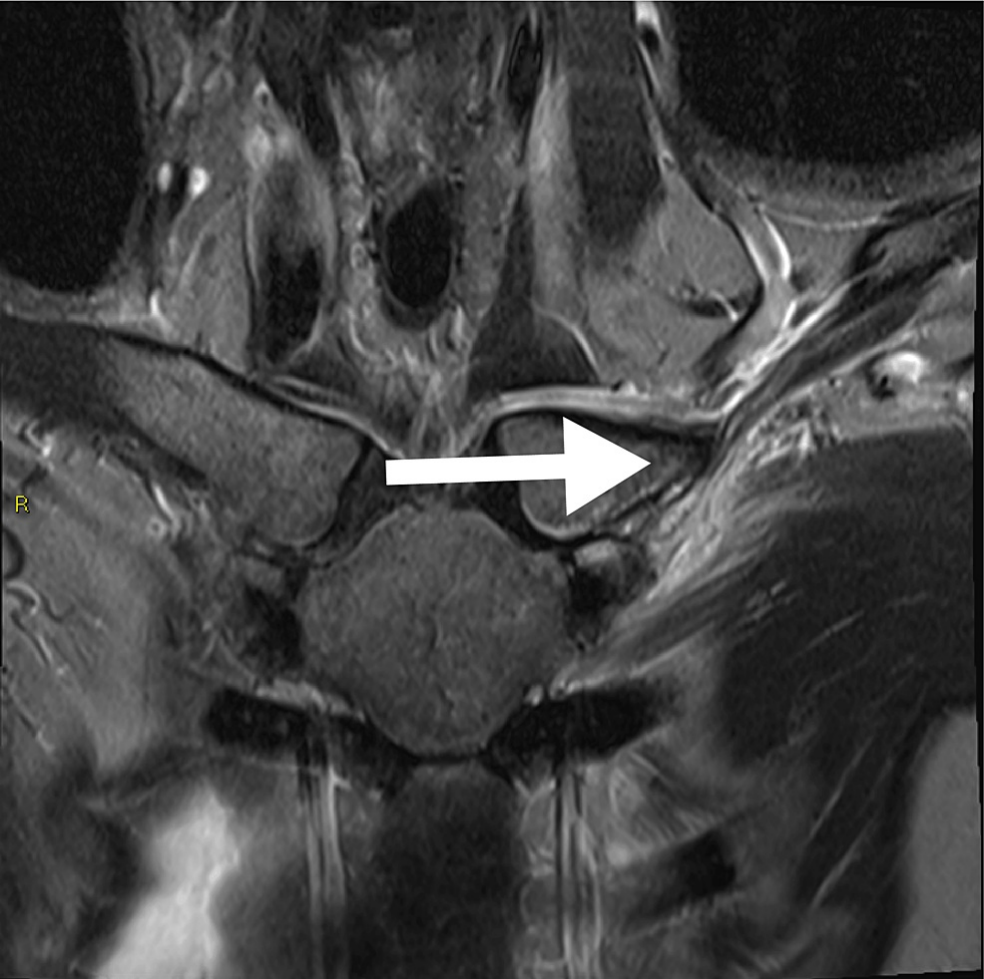
Coronal fat suppressed fluid sensitive MRI shows edema in the left distal first rib and surrounding soft tissue, in keeping with costochondritis. There is also mild signal change in the adjacent medical clavicle head. There is neither significant effusion of the left sternoclavicular joint, nor is there marrow edema in the sternum. The overlying pectoralis major muscle is edematous, likely secondary to reactive change.

However, in view of his significant pain, an MRI of the sternoclavicular joint was performed to rule out an abscess. It showed edema of his distal left first rib involving the costochondral junction. There was also mild edema of the left sternocostal joint, but much less so compared to the first costoclavicular joint. This was likely due to the close proximity between these locations. There was neither effusion nor significant marrow edema in the sternum (Figures 1-2). There was also enhancing edema in the slightly thickened left pectoralis major muscle and no abscess or fractures were seen. Diagnosis of Tietze’s syndrome post-COVID-19 infection was made.

The patient was started on nonsteroidal anti-inflammatory drugs (NSAIDs) ibuprofen 400 mg three times a day and also colchicine 500 mcg three times a day. The patient reported a reduction in pain, as well as an improvement in left shoulder range of movement with a good arm swing during ambulation. He was discharged on day four with both regular doses of colchicine and ibuprofen for another one week and as needed thereafter. Upon review in the clinic two weeks after, the patient reported a further improvement in his pain of about 80% of which ibuprofen was given as needed and another follow-up at week six revealed complete resolution of pain and swelling.

## Discussion

Tietze’s syndrome occurs due to inflammation of the costal cartilage which connects the ribs to the sternum. Patients frequently present with chest pain that is exacerbated with movement and/or positional changes. The pain tends to be exacerbated by movement and can be both dull and sharp in nature. Often, there is tenderness on palpation of the involved sternocostal joints of the chest wall [3]. Tietze’s syndrome normally affects single joints and is unilateral in 70% of the cases while costochondritis typically affects multiple joints and is bilateral in 90% of cases. Painful swelling of the affected area such as in this case helps to differentiate Tietze’s syndrome from costochondritis [4].

The exact cause is often unknown, although it has been associated with chronic excessive coughing, vomiting, trauma to the chest as well as respiratory infections particularly post viral. The etiology of chest pain determination in COVID-19 is crucial as lethal causes such as acute myocardial infarction and pulmonary embolism need to be excluded before committing to the diagnosis of Tietze’s syndrome or costochondritis [5]. The treatment generally involves the use of anti-inflammatory medications such as NSAIDs. Heat compression over the affected areas can also help to relieve pain. Colchicine was also successfully administered as an alternative to the standard therapy of NSAIDs to help resolve the pain associated with post-COVID-19 costochondritis in an 11-year-old boy [6]. In our case report, colchicine helped in addition to conventional analgesia such as NSAIDs and tramadol with good outcomes [7]. Although rarely needed, local administration of combined lidocaine/corticosteroid into costochondral areas can also be done in refractory cases in costochondritis and Tietze’s syndrome. Physiotherapy is crucial in improving symptoms and function caused by costochondritis [8].

To the best of our knowledge, this is the first published case of post-COVID-19 Tietze’s syndrome in an adult who is otherwise immunocompetent. Previously, post-COVID-19 costochondritis was reported in adolescents and adults treated with immunosuppressants [9-10]. However, there is no literature surrounding post-COVID-19 costochondritis or Tietze’s syndrome in an individual who had not recently received immunosuppressants. Nevertheless, the main limitation of this study is the inherent nature of a case report of an observation of Tietze’s syndrome after COVID-19, hence a causal relationship cannot be inferred from our study.

We recommend considering post-COVID-19 Tietze’s syndrome and costochondritis as differential diagnoses, especially if other causes have been ruled out in an individual with a recent COVID-19 infection. Early diagnosis and appropriate treatment can prevent repeated ED visits and improve patient care.

## Conclusions

This case report demonstrates a case of Tietze’s syndrome in a patient post-COVID-19 infection. The diagnoses of Tietze’s syndrome and costochondritis post-COVID-19 infection should be considered, after ruling out other sinister causes of chest pain. With increased awareness of post-COVID-19 complications, non-life-threatening conditions such as these may have a great impact on a patient’s quality of life. With appropriate diagnosis and prompt targeted treatment, repeated ED visits can be prevented with improved patient experience and reduced financial costs.
